# A multi-tool recipe to identify regions of protein-DNA binding and their influence on associated gene expression

**DOI:** 10.12688/f1000research.11616.2

**Published:** 2018-01-30

**Authors:** Daniel Carlin, Kassi Kosnicki, Sara Garamszegi, Trey Ideker, Helga Thorvaldsdóttir, Michael Reich, Jill Mesirov

**Affiliations:** 1 The University of California, San Diego School of Medicine, 500 Gilman Dr, La Jolla, CA, 92093, USA; 2 Broad Institute, Cambridge, MA, 02142, USA; 3 Moores Cancer Center, University of California, San Diego, La Jolla, CA, 92093, USA

**Keywords:** ChIP-seq, RNA-Seq, transcription factor, histone modification, epigenetics, regulation, differential expression, data integration

## Abstract

One commonly performed bioinformatics task is to infer functional regulation of transcription factors by observing differential expression under a knockout, and integrating DNA binding information of that transcription factor.   However, until now, this task has required dedicated bioinformatics support to perform the necessary data integration. GenomeSpace provides a protocol, or “recipe”, and a user interface with inter-operating software tools to identify protein occupancies along the genome from a ChIP-seq experiment and associated differentially regulated genes from a RNA-Seq experiment. By integrating RNA-Seq and ChIP-seq analyses, a user is easily able to associate differing expression phenotypes with changing epigenetic landscapes.

## Introduction

The genetic make-up of an organism plays a key role in gene regulation, especially during early cell differentiation and development. We can observe this phenomenon in siblings who possess different eye and hair color as a result of differing genetic code. However, epigenetic mechanisms, such as histone modifications, transcription factor binding and DNA methylation, also contribute to the complexity of individuals’ phenotypes as is observed in identical twins who possess the same genetic code while having slightly different features. Phenotypic differences associated with disease and varying stages of development have been mapped to changing patterns in gene regulation; and phenotype can often be attributed to a changing epigenetic landscape rather than hard-coded genetic features.

In order to decode these epigenetic differences, biologists often turn to an analysis based on two experimental assays; RNA sequencing (RNA-Seq) ( Nagalakshmi *et al.*, 2008; Wilhelm *et al.*, 2008), which quantifies the amount of (usually messenger) RNA in a cell, and Chromatin Immuno-precipitation sequencing (ChIP-seq) ( Johnson *et al.*, 2007; Robertson *et al.*, 2007), which shows where a particular protein binds the genome. Commonly, this protein is expected to have some influence on the mRNA expression of nearby genes (i.e., it is a transcription factor). Thus, by knocking out the gene that codes for the DNA binding protein and observing changes in mRNA expression, the biologist can infer the direct effect of the protein on expression.

When analyzing genomic data, today’s computational biologist may utilize a variety of different tools specific to each step of their analysis process. Not only must they be able to create the perfect marriage between the type of data and the tool, but they must be able to correctly manipulate the output, both for interpretation and for format conversion between tools. For the non-programming biologist, smooth integration of many of these tools is provided through GenomeSpace ( Qu *et al.*, 2016, www.genomespace.org) and its user-friendly “recipes” ( recipes.genomespace.org). GenomeSpace is a web-based visual workbench that supports a diverse range of bioinformatics tools and data resources popularly used in genomic analyses. Because GenomeSpace provides the ability to reformat data as it moves between software tools, one can create easy to use step-by-step workflows specific to a given analysis task. We refer to these published workflows as “recipes”.

We present one such recipe, currently available in GenomeSpace, which identifies differentially expressed genes between two samples, and compares that gene list with differential transcription factor occupancy from a ChIP-Seq experiment. This recipe is designed to elucidate which DNA-protein binding events are responsible for an observed change in mRNA expression. By identifying protein occupancies throughout the genome and comparing them to observed differences in mRNA expression, we can support hypotheses of functional regulation.

## Methods

**Figure 1.**
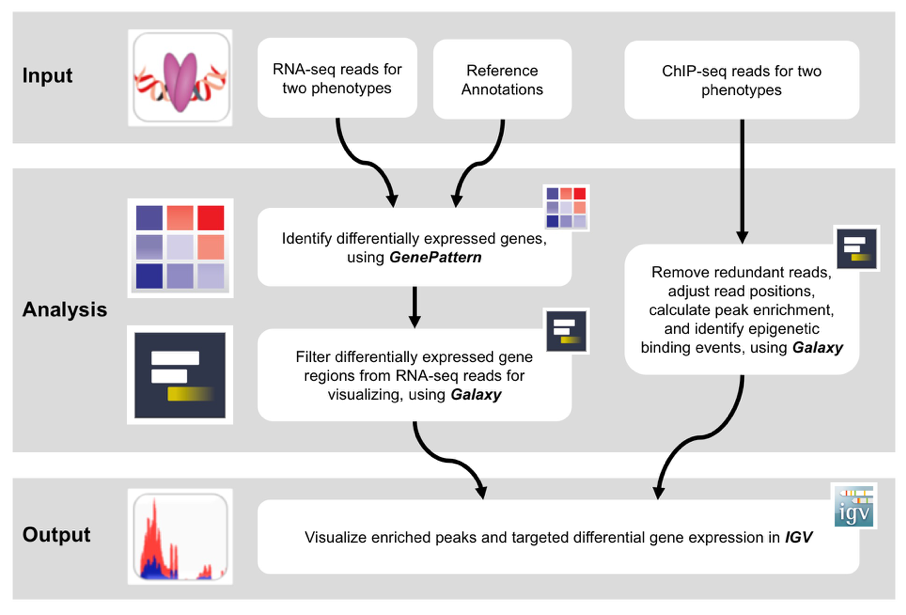
Workflow diagram.

This recipe takes as input the aligned reads from a differential RNA-seq transcription factor knockout experiment, and aligned reads from a ChIP-Seq experiment for the transcription factor that was knocked out. The output is a visualization of the genomic regions containing both differentially expressed genes and a binding site for the transcription factor. Since all tools used in this recipe are hosted remotely, running the recipe has no system requirements beyond an internet connection. An overall workflow diagram appears in Figure 1. We describe the individual steps of the recipe here.

### Obtaining and loading data

We start by obtaining a reference genome matching our model organism and aligning RNA-seq reads from two or more conditions (e.g. experimental and control) and ChIP-Seq reads from at least two samples, an input control and an experiment. In ChIP-Seq, the input control is a sample that has been run through all of the same preparatory and sequencing steps as the experiment, except for the antibody binding. This controls for the natural background of reads that are not selected by the binding of the target protein. Both RNA-seq and ChIP-Seq read data are uploaded to GenomeSpace in the BAM (Binary sequence Alignment MaP) format and the reference genome in the GTF (Gene Transfer Format). If users are beginning with unaligned reads, they can use a previously published RNA-seq recipe (http://recipes.genomespace.org/view/5) to pre-process their reads.

### Differential gene expression analysis

We next perform differential expression analysis using GenePattern ( Reich *et al.*, 2006, genepattern.broadinstitute.org), which can be launched from the GenomeSpace user interface. We use GenePattern’s *Cuffdiff* module to identify genes with differential expression between samples, measured by their FPKM (Fragments Per Kilobase of transcript per Million mapped reads) value. For each condition, we input the read data for an individual sample followed by the GTF reference genome. The output of the differential analysis is exported to GenomeSpace in *Cuffdiff’s* tabular format.

### Filtering and formatting differential gene expression data

We next launch Galaxy ( Afgan *et al.*, 2016; Giardine *et al.*, 2005, galaxyproject.org), again available through the GenomeSpace interface, and import RNA-seq reads from both conditions along with a file containing differential expression for each gene. This data is directly available through GenomeSpace. Using a Galaxy workflow, we filter genes that are significantly (q-value < 0.05) differentially expressed between the experiment (in this case a knockout) and control samples and extract their chromosome number, gene region start, gene region end, and gene symbol. Next we use Galaxy’s SAMtools ( Li *et al.*, 2009) *Filter* subtool, which extracts this data from the original RNA-seq reads in the BAM format. We convert the BAM files to the bigWig format so that they can be viewed in the Integrative Genomics Viewer (IGV) ( Robinson *et al.*, 2011; Thorvaldsdottir *et al.*, 2013).

### Identifying transcription factor binding sites

Next, we use GenomeSpace to import the ChIP-seq files from both the input control and experimental samples to Galaxy. Using Galaxy’s MACS2 ( Feng *et al.*, 2012) *callpeak* subtool, we obtain a bedGraph file containing peak data of both our experimental and input control files. Additionally, we use the MACS2 *callpeak* tool to identify differential peaks along the genome, indicative of transcription factor binding sites, and output this data as a bedGraph file. The two bedGraph files are converted in Galaxy to the bigWig format for visualization in IGV.

### Visualizing transcription factor binding sites and expression of associated genes

We next launch IGV through the GenomeSpace user interface. We select the appropriate reference genome included in IGV, and load all gene expression and peak-enrichment Bigwig files from GenomeSpace. Tracks are then scaled by group for visualization.

## Use case

**Figure 2.**
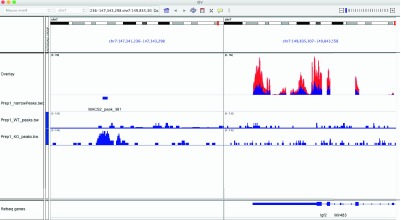
Epigenetic landscape of Prep1 binding and associated regulation of Igf2. The left panel illustrates the binding of the Prep1 transcription factor. In the right panel, we see the up-regulation of the gene, Igf2, as a result of this binding event.

We applied the recipe described above to an example dataset from Laurent *et al*. (2015), accession GSE6328, from NCBI’s Gene Expression Omnibus (GEO) database ( Barrett *et al.*, 2013; Edgar *et al.*, 2002). We can identify the interplay between the epigenetics and transcriptomics of mouse embryonic stems cells by observing how the binding of the transcription factor, *Prep1*, influences gene expression. *Prep1* is known for its contribution in embryonic development ( Laurent *et al.*, 2015). In comparing genome-wide maps of mouse embryonic cells expressing *Prep1* to those that do not, we can identify potential target genes that are being differentially regulated by these binding events. One such example of this is illustrated in Figure 2. Here, the transcription factor binding site has been identified and shown to up-regulate the expression of the gene *Igf2*.

## Variations of this recipe

This recipe can be used, not only to identify the regulation of genes by transcription factor binding, but also to identify any epigenetic mechanism that can be analyzed by ChIP-sequencing. For example, we can identify regions in the genome where histone modifications have occurred, and match those regions to observed changes in expression presumably resulting from the histone modifications. However, we must consider the nature of the data when selecting parameters in the MACS2 tool in Galaxy. For example, when performing peak enrichment on histone modification occupancies, a user must select an advanced option to include broader regions, since histone modifications are represented by a much broader peak area along the genome.

The GenomeSpace recipes use the tools with which the authors are most familiar and comfortable. However, we do recognize that there are alternative tools and approaches to completing the same analysis. For instance, DESeq2 ( Love *et al.*, 2014) can be employed in Gene Pattern as an alternative method for calling differential expression. Furthermore, there are many other alternatives for calling differentially expressed genes though they may not be available within the family of GenomeSpace tools, e.g., EdgeR ( Robinson *et al.*, 2010), and so any issues of format compatibility would have to be handled by the user.

We note that CuffDiff ( Trapnell *et al.*, 2013) and MACS2 are available through Galaxy in addition to GenePattern, and can be employed through the framework if the user is more comfortable with the Galaxy tool.

## Data availability

The original ChIP-seq and RNA-seq data of this experiment have been deposited in GEO, with accession number GSE63282. The recipe providing all the detailed steps and corresponding videos associated with this process is accessible at: http://recipes.genomespace.org/view/69.
