# Supplementary material for: A multi-tool recipe to identify regions of protein-DNA binding and their influence on associated gene expression
Source: F1000Res. 2018 Jan 30;6:784. Originally published 2017 Jun 6. [Version 2] doi: 10.12688/f1000research.11616.2 (PMC5806050; doi:10.12688/f1000research.11616.2)
Supplement: Peer review history

# Peer review: Referee response for version 2

**DOI:** 10.5256/f1000research.14929.r30399

**Published:** 2018-02-07

**Authors:** Isha Sethi

**Affiliations:** 1 Dana Farber Cancer Institute & Harvard School of Public Health, Boston, MA, USA

The authors have mostly addressed the  reviewers concerns. However, one minor point: the authors still mention the peaks called by MACS2 as differential peaks. As reviewer 1 explains, this is misleading. Differential peaks are those derived by comparison between 2 experiments, not whats MACS does (that is calculating enrichment in experiment over input).

I have read this submission. I believe that I have an appropriate level of expertise to confirm that it is of an acceptable scientific standard.

# Peer review: Referee response for version 1

**DOI:** 10.5256/f1000research.12547.r24958

**Published:** 2017-08-22

**Authors:** Andrew D Sharrocks, Munazah Andrabi

**Affiliations:** 1 Faculty of Life Sciences, University of Manchester, Manchester, UK

The authors present a workflow (which they refer to as a “recipe”) for the integration of RNA-Seq and ChIP-Seq experiments to find associations between genomic binding of TFs and their potential direct effects on the mRNA expression using the web-based work-bench GenomeSpace. The workflow enables integration of differential gene expression analysis following transcription factor knockdown (RNA-seq data) with binding data for the same transcription factor (ChIP-seq data). While there are many useful pipelines available for analysing and integrating sequencing data, GenomeSpace and its associated “recipes” make the analysis and integration of the data less daunting for a biologist with little or no programming experience.

 Overall this “recipe” will likely be useful for biologists. However, we would like to make a few comments:

 1. The authors do not make it clear whether the user has to pre-align the files to the reference genome before uploading them to GenomeSpace or the alignment itself can be done via their recipe. If not then it will be useful to provide the necessary tools and guidance required for alignment given that aligning the reads to genome is one of the most memory and time consuming steps.

 2.The authors use the Cuffdiff module from the Cufflinks package to perform differential expression. Although the goal of this recipe is providing user-friendly and simplified workflow for integration of data the authors should mention the advantages of alternate tools such as EdgeR and DeSeq for identifying differential expression. These tools are available in GenePattern therefore it will be sensible to provide the user with all options. Especially since these tools are known to have  better normalisation techniques and perform a more robust and reliable identification of differentially expressed genes compared to Cuffdiff.

 3. The authors should not confuse the *Enriched peak* data obtained by using the MACS callpeak tool on the ChIP data and its input with *Differential peak*s. Differential peaks are obtained between two experimental conditions and not between the ChIP experiment and its input. This language can be misleading especially for beginners.

 4. A flowchart of the analysis steps in the paper would be highly useful to get started.

 5. Figure 1 is not entirely clear as presented. It is not clear why two separate panels are provided rather than a single panel that shows the location of the ChIP-seq peaks relative to the gene expression changes. Also, indicating what the colours represent in the gene expression data. The track labelling on the left is also not clear, and presumably “overlay” is the RNAseq data and “peaks” the ChIP-seq data. While the current labelling is presumably driven by the naming of the original files, better labelling is suggested in the context of this figure so it is clear to the reader.

 Finally, looking at the coordinates provided, we are not convinced that this is a good example to provide. The TF binding event Appears to be over 2.5Mb from the TSS of the putative target gene, meaning that any links here would be fairly low confidence. This would of course become more obvious if displayed on a single panel.

 6. To make the “recipe” really useful, it would be good to have outputs beyond simple genome browser views. Having a tabular output of differentially expressed genes and the relative location(s) (and coordinates) of any binding peaks for the transcription factor in question would be useful to have.

We have read this submission. We believe that we have an appropriate level of expertise to confirm that it is of an acceptable scientific standard.

# Peer review: Referee response for version 1

**DOI:** 10.5256/f1000research.12547.r23943

**Published:** 2017-07-11

**Authors:** Isha Sethi

**Affiliations:** 1 Dana Farber Cancer Institute & Harvard School of Public Health, Boston, MA, USA

    The authors have made a pipeline integrating differential RNA-Seq expression analysis with ChIP-Seq analysis and implemented it through the GenomeSpace platform. Though as mentioned by the authors in the paper: this is a commonly performed bioinformatic task, their aim is to make this integrated analysis easily accessible to non-bioinformatic users. For this purpose their workflow on the web-based workbench involves integrating multiple tools like Cuffdiff module in GenePattern (for Differential RNA-Seq analysis) with  MACS2 in Galaxy (for ChIP-Seq analysis). 

         Though the workflow presented by the authors seems easy to use by any biologist, it also appears to be severely limited not just in its scope of application but also in its choice of tools which are hardcoded. For example the authors use "CuffDiff" for Differential RNA-Seq expression analysis. The authors do not state why they chose this particular method or even why they chose its GenePattern module and not the Galaxy implementation. Though admittedly this is a popular tool and has the advantage of transcript level analysis, it also suffers from known limitation of underestimating the number of differential genes. Other count based method like DESeq2 tool (also implemented in Galaxy) might be better suited for most gene-level differential RNA-Seq analysis. Also, the authors do not clearly explain why their workflow is better or easier for a biologist to implement than using the same tools through Galaxy directly (which has been made for a non-coding biologist).  I would argue that working directly on Galaxy even if slightly more complicated would be more rewarding to users as it offers not just greater flexibility of tools but also the option to select different parameters than default. 

      Hence in conclusion, to make this manuscript better the authors should 1) provide a clearer explanation for their choice of tools and why is it easier/better to use their pipeline than the same tools on Galaxy directly, 2) If possible the authors should try to expand their workflow to provide a greater flexibility to the user to choose their tools for RNA-Seq and ChIP-Seq analysis.

I have read this submission. I believe that I have an appropriate level of expertise to confirm that it is of an acceptable scientific standard, however I have significant reservations, as outlined above.

# Response

**Published:** 2017-09-27

**Authors:** Jill Mesirov

**Affiliations:** Department of Medicine, University of California, San Diego, USA

We greatly appreciate the reviewers' comments  pointing out that the "Variations of the Recipe" section of this manuscript did not discuss alternative tools.  The GenomeSpace recipes are subjective in that they use tools with which the authors are most familiar and comfortable.  However, we do recognize that there are, of course, other options. We will revise the manuscript (and the recipe) to include some of the other options that could be used.  In the past that has been our custom and we apologize that we did not catch the lack of this information in this recipe.  In fact, on the best practices page of our website - http://recipes.genomespace.org/page/best-practices-for-creating-recipes we suggest authors supply alternatives. We would invite both the reviewers and other readers of the manuscript to contribute versions of this recipe using their preferred tools and approaches. GenomeSpace is a community project and contribution of recipes is both welcome and encouraged.
